# Herb Target Prediction Based on Representation Learning of Symptom related Heterogeneous Network

**DOI:** 10.1016/j.csbj.2019.02.002

**Published:** 2019-02-08

**Authors:** Ning Wang, Peng Li, Xiaochen Hu, Kuo Yang, Yonghong Peng, Qiang Zhu, Runshun Zhang, Zhuye Gao, Hao Xu, Baoyan Liu, Jianxin Chen, Xuezhong Zhou

**Affiliations:** a School of Computer and Information Technology and Beijing Key Lab of Traffic Data Analysis and Mining, Beijing Jiaotong University, Beijing 100044, China; b College of Arts and Sciences, Shanxi Agricultural University, Taigu 030801, China; c Faculty of Computer Science, University of Sunderland, St Peters Campus, Sunderland SR6 0DD, UK; d Medical Intelligence Institute, School of Computer and Information Technology, Beijing Jiaotong University, Beijing 100044, China; e Guanganmen Hospital, China Academy of Chinese Medical Sciences, Beijing 100053, China; f Department of Cardiology, Xiyuan Hospital of China Academy of Chinese Medical Sciences, Beijing 100091, China; g Data Center of Traditional Chinese Medicine, China Academy of Chinese Medical Sciences, Beijing 100700, China; h Beijing University of Chinese Medicine, Beijing 100029, China

**Keywords:** Network medicine, Herb target prediction, Symptoms, Network embedding

## Abstract

Traditional Chinese Medicine (TCM) has received increasing attention as a complementary approach or alternative to modern medicine. However, experimental methods for identifying novel targets of TCM herbs heavily relied on the current available herb-compound-target relationships. In this work, we present an Herb-Target Interaction Network (HTINet) approach, a novel network integration pipeline for herb-target prediction mainly relying on the symptom related associations. HTINet focuses on capturing the low-dimensional feature vectors for both herbs and proteins by network embedding, which incorporate the topological properties of nodes across multi-layered heterogeneous network, and then performs supervised learning based on these low-dimensional feature representations. HTINet obtains performance improvement over a well-established random walk based herb-target prediction method. Furthermore, we have manually validated several predicted herb-target interactions from independent literatures. These results indicate that HTINet can be used to integrate heterogeneous information to predict novel herb-target interactions.

## Graphical Abstract

**Figure:**
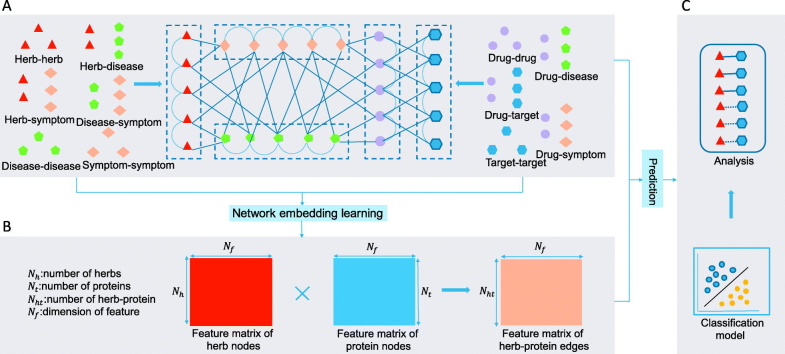

## 1 Introduction

The post-genome era argues a far more complex landscape of disease that complex biological processes driving human disease are nearly always the integrative result of multiple pathways that interact through an interconnected network and even spread across most of the genome [1,2]. This complexity puts forward a great challenge to conventional medicine that develops drugs focusing on a single molecular effector to treat disease. Alternatively, Traditional Chinese Medicine (TCM) has received increasing attention as a complementary approach or alternative to modern medicine for its poly-pharmacological effects [3,4].

A TCM prescription is a mixture of several herbs that contain numerous chemical ingredients acting on different pharmacological targets and regulating multiple biological mechanisms. Although promising, the complexity of TCM makes it impossible to parse the underlying mechanisms using the reductionist method of identifying one active ingredient to hit one biological target. Most TCM herbs contain dozens to thousands of ingredients, and only a fraction is effective. Experimental methods for identifying novel targets of TCM herbs need to both identify active ingredients and the corresponding biological targets, which can be extremely costly and time-consuming [5,6].

In recent years, researchers have developed a series of systems or network pharmacological strategies to detect the molecular mechanisms of TCM [7]. These studies adopt the strategy of “herb to ingredient to target”: firstly, collect the TCM ingredients, and then the potential targets of these ingredients are identified on a proteome-wide scale with in silico ligand-based target prediction approaches, following in vivo validation. These studies have provided a more comprehensive understanding of the pharmacological basis of TCM [8,9]. However, the performance of this strategy is also limited by the biochemical features of TCM and the drawback of the ligand-based approaches: for example, there are still many components that are undiscovered for some TCM. The ligand-based approaches often lead to poor prediction results when a target has only a small number of known binding ligands.

Inspired by these considerations, we expected to develop a computation method that could explore the mechanisms of TCM by avoiding the incorporating of the chemical compositions of herbs. Recently, Tao et al. [10] presented a novel integrative approach combining ontology reasoning with network-assisted gene ranking to predict new drug targets. Yuan et al. [11] proposed the DrugE-Rank to improve the prediction performance by combining the advantages of the feature-based and similarity-based methods. Zong et al. [12] described a similarity-based drug-target prediction method that utilizes a topology-based similarity measure and two inference methods based on the similarities. Zhang et al. [13] investigated the pharmacological mechanisms of Wu-tou decoction acting on rheumatoid arthritis through systems approaches integrating drug target prediction, network analysis and experimental validation. In addition, network-based methods have been introduced to explore the latent correlation features of drug-target interactions to predict new interactions [14]. A key feature of network-based approaches is to incorporate heterogeneous data including chemical, pharmacological, genomic, functional or phenotypic data to boosting the accuracy of drug-target prediction [15]. The network-based methods have also been successfully used for herb-target prediction. For example, Vanunu O et al. proposed PRINCE method [16], which was originally used in disease-gene prediction, and then was introduced into TCM target prediction by Yang et al. [17]. Meanwhile, according to the “network pharmacology”, the “one target, one drug” paradigm was shifted to the “network target, multi-component” strategy with the rapid progress in bioinformatics and polypharmacology [18]. In TCM network pharmacology, target profile prediction is one of the critical procedures [19]. A computational framework, drugCIPHER, had been developed to achieve this goal [20], which was used to predict target profiles of herbal compounds by searching for new potential uses for known bioactive compounds. Also, Yang et al. proposed a network-based method [21] using the random walk to detect novel herb-target interactions. However, these approaches solely relied on the current available herb-compound-target relationships, which have significant bottlenecks for the herb target prediction since the herb-target relationships are in high noise rate and incomplete.

In this paper, we present an Herb-Target Interaction Network (HTINet) approach, a novel network integration pipeline for herb-target prediction mainly relying on the symptom related associations. Our method applies a network-embedding algorithm, called node2vec [22], to encode the heterogeneous network associated with herbs and targets by interconnecting the phenotypes (i.e. symptoms and diseases) to both herbs and proteins. In addition, HTINet not only integrates diverse information from heterogeneous data sources (e.g., herbs, symptoms, diseases, drugs, and proteins), but also copes with the noisy, incomplete and high-dimensional nature of large-scale biological data by learning low-dimensional but informative vector representations for both herbs and proteins. The low-dimensional feature vectors learned by HTINet capture the context information of individual networks, as well as the topological properties of nodes (e.g., herbs or proteins) across multi-layered heterogeneous network. Then, these low-dimensional vectors could be used as feature representations of nodes for the next step of supervised learning. A series of classical supervised learning models including K-Nearest Neighbor (KNN), Support Vector Machines (SVM), Logistic Regression (LR), Decision Tree (DT), Random Forest (RF) and Gradient Boosting Decision Tree (GBDT) were tested in our work. We compared the performance of HTINet with other herb-target prediction methods. Furthermore, we have validated some interactions predicted by HTINet between three herbs and their protein targets curated in TCMID database.

## 2 Materials and Methods

### 2.1 Data Acquiring and Processing

We obtained the heterogeneous information covering the herb, symptom, disease, drug and target and their corresponding interactions from various public databases and publications (Table S1). Briefly, the herb-target relationships were obtained from HIT database, and regarded as the standard data set for training and evaluation of the classification model. The relationships between herbs and indications were collected from Chinese pharmacopoeia (CHPA, 2015 edition). The drug-indication relationships were extracted from SIDER [23]. The diseases-symptom relationships were collected from MalaCards database [24]. The drug-target relationships were collected from DrugBank [25].

The herb-herb associations were built following our previous work [21]. Specifically, the herb-efficacy associations were collected from CHPA. According to the relationships of herb-efficacy, we built the efficacy-based herb vectors. Then the efficacy-based cosine similarities of herb pairs were calculated, which form the herb-herb associations, with cosine similarity as edge weight. For example, there were *m* types of herbs and *n* types of efficacies, and each herb *a* can be represented by a vector of efficacy *V*_*a*_ = (*w*_*i*_, *w*_2_,  … , *w*_*j*_,  … , *w*_*n*_), where *w*_*j*_ = 1 if herb *a* has relationship with efficacy *j*, if not, *w*_*j*_ = 0. Then the efficacy-based cosine similarity of herb *a* and *b* can be calculated by Eq. (1).

(1) $$\mathrm{Cos}\left(V_{a},V_{b}\right)=\frac{V_{a}∙V_{b}}{\left|V_{a}\right|∙\left|V_{b}\right|}$$

The disease-disease and symptom-symptom relationships were investigated by text mining techniques. Firstly, both disease-disease, symptom-symptom relationships were extracted from the Semantic MEDLINE Database (SemMedDB) [26], which contains all (subject, predicate, object) ternary semantic relationships extracted from the MEDLINE database by the biomedical Semantic relation extraction tool SemRep [27]. Then the significance of each relationship was calculated by the Fisher's exact test [28]. Finally, those significant relationships (*P* < .05) through the inspection of clinicians were considered as reliable data. To further analysis the disease-disease associations, we calculated the number of the common nodes and links with the HSDN disease network in Zhou et.al [29]. The results shown that the disease-disease associations in HTINet captures 2241 common nodes and 6153 common links (33.4% of the whole disease-disease associations in HTINet, 99.0% of the subnetwork of the 2241 common nodes in HTINet). The *P*-Value of the observed number of 6153 shared links can be computed from the binomial distribution to be *P* = 1.8 × 10^−21^, indicating that the HTINet offers reliable disease-disease associations.

The drug-drug associations were built by linking drugs with similar ATC (Anatomical Therapeutic Chemical) classifications. The drug ATC classification tree was obtained from KEGG database [30]. The ATC classification tree records the functional classification of drug in a tree-like structure. For each drug, we can build a vector based on category encoding. For example, there were *m* types of drugs and *n* types of ATC categories, and each drug *a* can be represented by a vector of ATC categories *V*_*a*_ = (*w*_*i*_, *w*_2_,  … , *w*_*j*_,  … , *w*_*n*_), where *w*_*j*_ = 1 if drug *a* belonging to category *j*, if not, *w*_*j*_ = 0. Then the ATC-based cosine similarities between the relationships of two drugs can be measured by Eq. (1).

The protein-protein interactions were extracted from the popular gene-gene interaction network database, Search Tool for Recurring Instances of Neighboring Genes (STRING) [31], which quantitatively integrate different studies and interaction types into a single integrated score for each gene pair based on the total weight of evidence. We filtered the relationships with a weights >700 [29,32], and made linear normalization (Eq. (2)), also known as min-max normalization, which is a linear transformation of the original data, so that the weight is mapped to [0, 1].

(2) $$x^{\prime }=\frac{x-\min\left(x\right)}{\max\left(x\right)-\min\left(x\right)}$$

### 2.2 Construction of the Heterogeneous Network

**Fig. 1:**
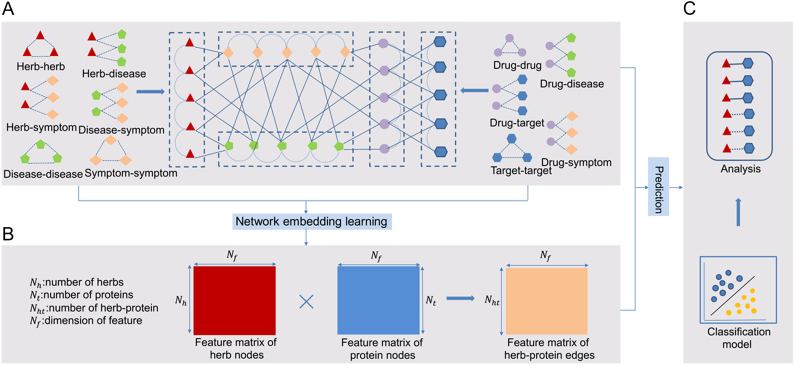
The overview of HTINet method. (A) HTINet firstly builds a heterogeneous network by integrating 5 types of nodes and corresponding 11 types of edges from diverse datasets (see details in Materials and Methods). (B) Secondly, the latent representations of herb and protein nodes were extracted in the heterogeneous network using an embedding learning algorithm node2vec. And the feature matrix of herb-protein interactions was constructed by Hadamard product using the feature vectors of all herb and protein nodes. (C) Finally, the classification models were constructed to predict the herb-target interactions.

We constructed the heterogeneous network (Fig. 1A) using 5 types of nodes (i.e., 2274 herbs, 3730 symptoms, 19,454 diseases, 2148 drugs and 15,709 protein targets) and 11 types of associations including 15,133 herb-herb associations, 476 symptom-symptom associations, 18,418 disease-disease associations, 4481 drug-drug associations, 319,889 protein-protein interactions, 7982 herb-symptom associations, 23,451 herb-disease associations, 72,248 disease-symptom associations, 4864 drug-symptom associations, 12,821 drug-disease associations and 7077 drug-target interactions (Table S1).

### 2.3 Network Embedding

In recent machine learning on graphs and networks, embedding is a novel method to learn latent representation for nodes in network [33]. The latent representation also is known as a *d*-dimensional vector representing the feature of node by capturing a network's structural properties. Here *d* is a parameter selecting the number of dimensions of learning feature representation. The output feature representations can be used as the input of machine learning algorithms for various network science tasks, such as classification and link prediction [34].

The previous researches with networks usually considered network structural properties, such as betweenness centrality and modularity, which required inclusive domain knowledge and expensive computation. Dealing with these issues, network embedding has been extensively studied in order to automatically discover and map a network's structural properties into a latent space [33]. In addition, the main challenge in biology is finding the feature to represent terms in biology network so that it can be easily utilized by machine learning models. Traditionally, simple chemical sub-structure and sequence order information of drug and target were chosen as the drugs and targets representation, which need a lot of molecular information and dimension of feature is high [35]. But there is no need for network embedding to get herbal compound and molecular information to represent herb and target, and embedding could obtain the low-dimensional feature.

#### 2.3.1 Node2vec

Node2vec, a type of embedding method, is used as an algorithmic framework for learning feature representations for nodes in the networks [22]. Compared to the previous embedding method (e.g., LINE [36], DeepWalk [37]), a biased random walk strategy was used in node2vec, which can flexibly and efficiently explore the diverse neighborhoods of nodes [38]. In the framework, *G* = (*V*, *E*) is an input network with *V* as the node set and *E* as the edge set. Let *f* : *V* → *R*^*d*^ be the mapping function from nodes to feature representations used for a next prediction task. The node2vec mainly includes the following two aspects.

##### 2.3.1.1 Random Walks

When node2vec learns the vector representation for nodes in *V*, it first simulates a random walk to sample neighborhoods of the source node *u*. Let *c*_*i*_ denote the *i*th node in the walk, starting with *c*_0_ = *u*. Nodes *c*_*i*_ in the walk are generated by the following distribution:

(3) Pci=xci−1=v,ci−2=t=πvxZ,ifvx∈E0,otherwise

where *π*_*vx*_ = *α*(*t*, *x*) ∙ *w*_*vx*_ is the unnormalized transition probability between nodes *v* and *x*, and *w*_*vx*_ represents the edge weight between nodes *v* and *x*, and *Z* is the normalizing constant. Let *α*(*t*, *x*) be calculated by following:

(4) αtx=1pifdtx=01ifdtx=11qifdtx=2

where *d*_*tx*_ is the shortest path between nodes *t* and *x*. Thus, it can guide the random walks process with two parameters *p* and *q*. If we set parameter *p* is a high value, the walk is less likely to sample already-visited nodes. On the other hand, if *q* is high, it is biased to sample nodes that are close to node *u*.

##### 2.3.1.2 Feature Learning

Let *N*_*S*_(*u*) ⊂ *V* be defined as a network neighborhood of node *u* generated through a random walk. Then node2vec learns feature representations by maximizing the likelihood of preserving node neighborhoods (Eq. (5)), and the feature representation for a node *u* is a *d*-dimensional vector, where *d* may be chosen by the user.

(5) $$\max_{f}\;\sum_{u\in V}\left[-\log\sum_{v\in V}\exp\left(f\left(u\right)∙f\left(v\right)\right)+\sum_{n_{i}\in N_{S}\left(u\right)}f\left(n_{i}\right)∙f\left(u\right)\right]$$

Therefore, we learn a mapping of nodes to a low-dimensional space of features that maximizes the likelihood of preserving network neighborhoods of nodes in node2vec.

#### 2.3.2 Learning Edge Features

In our work, we are interested in predicting the relationships between herbs and targets. Since we have got the feature representations of herbs and targets from node2vec, we extend them to pairs of herbs and targets using a bootstrapping approach over the feature representations of the individual node. Given two nodes herb *u* and target *v*, we define the relationship between herb-target using the Hadamard product [22] of feature vectors *f*(*u*) and *f*(*v*) (Eq. (6)).

(6) $$f\left(u,v\right)=f\left(u\right)⊙f\left(v\right)$$

Thus, we get the herb-target relationship from previous process. Even if an edge of herb and target does not exist since doing so makes the representations useful for our next classification model prediction task. According to the node2vec algorithm, the vector representation of nodes can be considered as the feature of nodes. In this way, the feature representation of herb-target relationship is obtained through the feature representation of nodes, which is used as the training and test of classification.

### 2.4 Machine Learning Model

The output feature representations from embedding learning can be used as the input of machine learning tasks. These features are applied to the task of herb-target edge prediction, in which we intend to estimate the probability that the herb-target edge according to their vector representation. Six classic supervised learning methods implemented in the sklearn library [39] including the K-Nearest Neighbor (KNN), Support Vector Machines (SVM), Logistic Regression (LR), Decision Tree (DT), Random Forest (RF) and Gradient Boosting Decision Tree (GBDT) were used to train the model.

## 3 Result

### 3.1 Overview of HTINet

We developed a new method called HTINet to predict herb-target interactions from a heterogeneous network. As an overview (Fig. 1), HTINet firstly builds a heterogeneous network by integrating 5 types of nodes, and the corresponding 11 types of edges from diverse datasets (see details in Materials and Methods). Totally, the heterogeneous network includes 43,315 nodes and 486,840 edges. Subsequently, we extracted the latent representations of herb and protein nodes in the heterogeneous network using a network embedding algorithm node2vec [22]. The algorithm combines a random walk process and a shallow neural network with one hidden layer to capture the underlying structural properties of the heterogeneous network. After getting the feature vectors of all herb and protein nodes in the heterogeneous network, we constructed the features of herb-protein interactions by Hadamard product [22] of the feature of each herb and protein node. In this way, we obtained a large-scale feature matrix with 12,520,083 herb-protein interactions that pack the heterogeneous network information led by the corresponding herb and protein nodes. Based on the feature matrix of the herb-protein interactions, those classical supervised learning models including K-Nearest Neighbor (KNN), Support Vector Machines (SVM), Logistic Regression (LR), Decision Tree (DT), Random Forest (RF) and Gradient Boosting Decision Tree (GBDT) can be applied to construct classification models to predict the herb-target interactions.

### 3.2 Performance of Models

In our model, we selected the HIT database [40], a comprehensive and fully curated database of herbs and their corresponding protein targets, as the benchmarks for herb-target interactions containing 8933 herb-target interactions. A 10-fold cross-validation procedure was utilized to evaluate the performance of HTINet. Specifically, the benchmark data were randomly divided into 10 subsets. Then the 9 subsets and a matching number of randomly sampled non-interacting interactions were selected as the training dataset to train a classification model. The remaining one subset and a matching number of randomly sampled non-interacting interactions were used as testing dataset. This cross-validation process was repeated ten times. Cross-validation is used to prevent over-fitting caused by the model. Using 10-fold cross-validation, all samples were used as training and test sets, and each sample was verified once, avoiding wasted data. A large number of experiments using a large number of data sets and using different learning techniques showed that 10% is the right choice for obtaining the best error estimate.

**Fig. 2:**
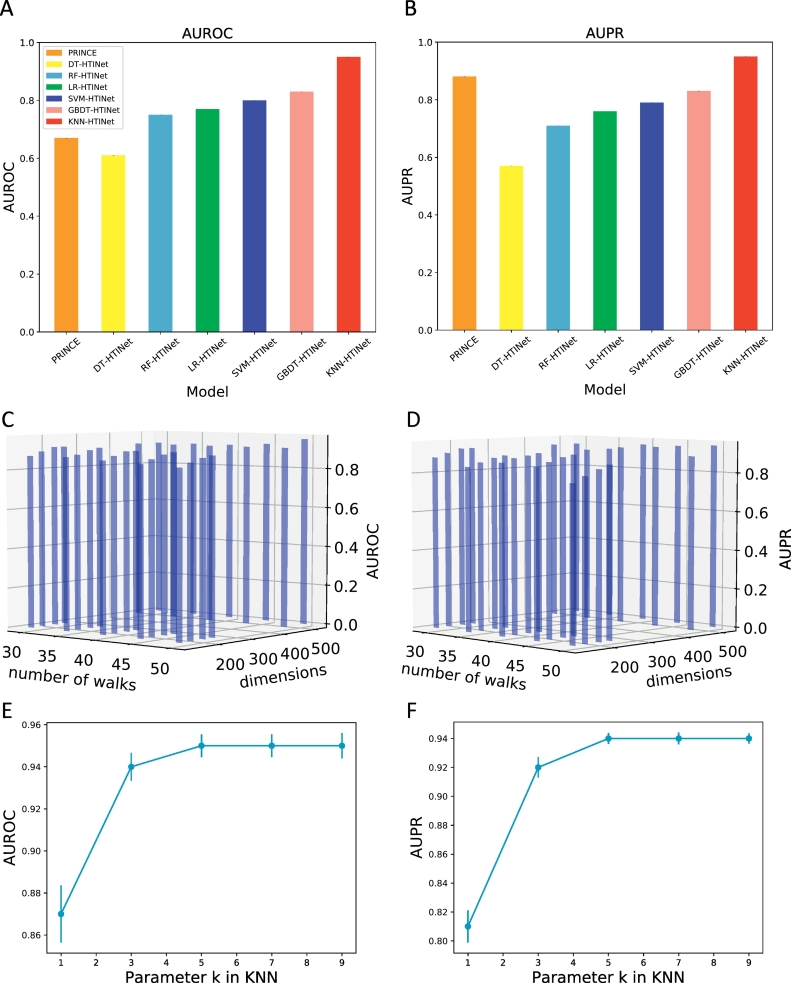
The performance of PRINCE and HTINet for herb-target interaction prediction and parameters adjustment of HTINet. A 10-fold cross-validation procedure was used to evaluate the performance of PRINCE, DT-HTINet, RF-HTINet, LR-HTINet, SVM-HTINet, GBDT-HTINet and KNN-HTINet. The area under the ROC curve (AUROC) and area under the precision-recall curve (AUPR) were used to evaluate the performance of the model. **(A)** The AUROC distribution of the models. **(B)** The AUPR distribution of the models. **(C)** The AUROC distribution under different number of walks and dimensions. **(D)** The AUPR distribution under different number of walks and dimensions. **(E)** The AUROC distribution under different parameter *k*. **(F)** The AUPR distribution under different parameter *k*.

In this work, we chose six classical supervised learning models KNN, SVM, LR, DT, RF and GBDT to examine the performance of HTINet. Both area under the receiver operating characteristic (ROC) curve (AUROC) and area under the precision-recall curve (AUPR) were used to evaluate the performance of the model (Table S2). As shown in Fig. 2A, B, DT-HTINet is the worst-performing model with both AUROC (61%) and AUPR (57%). The more sophisticated model RF-HTINet, LR-HTINet, SVM-HTINet and GBDT-HTINet perform better, achieving an AUROC of 75%, 77%, 80% and 83% and an AUPR of 71%, 76%, 79% and 83%, respectively. More importantly, we found that KNN-HTINet achieves an AUROC of 95% and an AUPR of 94%, a clear improvement in prediction performance over other HTINet models. Meanwhile, many common artificial intelligence algorithms are inspired by nature, including artificial neural networks which have been designed and built to mimic brain function on a neuron level. Neural Networks can be used to solve a wide range of problem types including both regression and classification problems. Therefore, we also tested the performance of model using the artificial neural networks, obtaining an AUROC of 82% and an AUPR of 79%. In addition, we also tested the performance of the HTINet models whose drug-drug similarities were calculated by Jaccard similarity (Table S3). The results indicated that the KNN-HTINet still achieved a clear improvement in prediction performance over other HTINet models, with an AUROC of 91% and an AUPR of 93%. But the performance is lower than KNN-HTINet whose drug-drug similarities are calculated by cosine similarity, with an AUROC of 95% and an AUPR of 94%. Thus, the KNN-HTINet model whose drug-drug similarities are calculated by cosine similarity is used in the following analysis.

Furthermore, we compared the prediction performance of HTINet with PRINCE method, which is an iteration algorithm spreading information of known nodes to other nodes in the network. HTINet outperforms PRINCE method, with 28% higher AUROC (67%) and 6% higher AUPR (88%). As previous studies [41,42] indicated, ROC is an overoptimistic metric to evaluate the performance of an algorithm in prediction tasks, especially on highly-skewed data. Comparatively, PR model present a better assessment in this scenario. The noticeable performance improvement of KNN-HTINet in terms of AUPR over PRINCE demonstrated its superior ability in predicting novel herb-target interactions in the heterogeneous networks. This could be explained from their principles. PRINCE starts with the target nodes to capture other nodes with similar information, which should be effective for extracting local features in uniformly distributed networks rather than the heterogeneous network with various types of sparse data. Alternatively, HTINet utilized a combined random walk and neural network-based feature learning procedure, which can integrate more domain information to learn much broader and deeper node information in the heterogeneous network. After comparing the AUC and AUPR between HTINet and PRINCE, we also calculated the Wilcoxon signed-rank test (*p*-value is 0.000295) according to the results of PRINCE and HTINet. The results indicated a significant difference in results of PRINCE and HTINet.

### 3.3 Parameters Adjustment of HTINet

In HTINet, the node2vec was used to get the feature vector representation of nodes in network. We need to learn two important parameters: number of walks and embedding dimensions. We adjusted the parameters of the algorithm by evaluating the performance of HTINet under different parameters. Specifically, embedding dimensions were test under the following parameters: 128, 160, 196, 224, 448 and 512, and number of walks: 30, 35, 40, 45 and 50. We found the best performing parameters to be 512 for the embedding dimensions, and 50 for the number of walks, and we fix these parameters throughout our experiments (Fig. 2C, D). In addition, the value of *k* in KNN prediction model is also important, we evaluated the performance of HTINet under different *k*, which include 1,3,5,7 and 9. We found that KNN model had the best performance when parameter *k* is 5, 7 and 9 (Fig. 2E, F). In our experiments, we chose 5 as value of *k*, as other two *k* values (7 and 9) would increase the approximate error.

### 3.4 Prediction of Novel Herb-Target Interactions

**Fig. 3:**
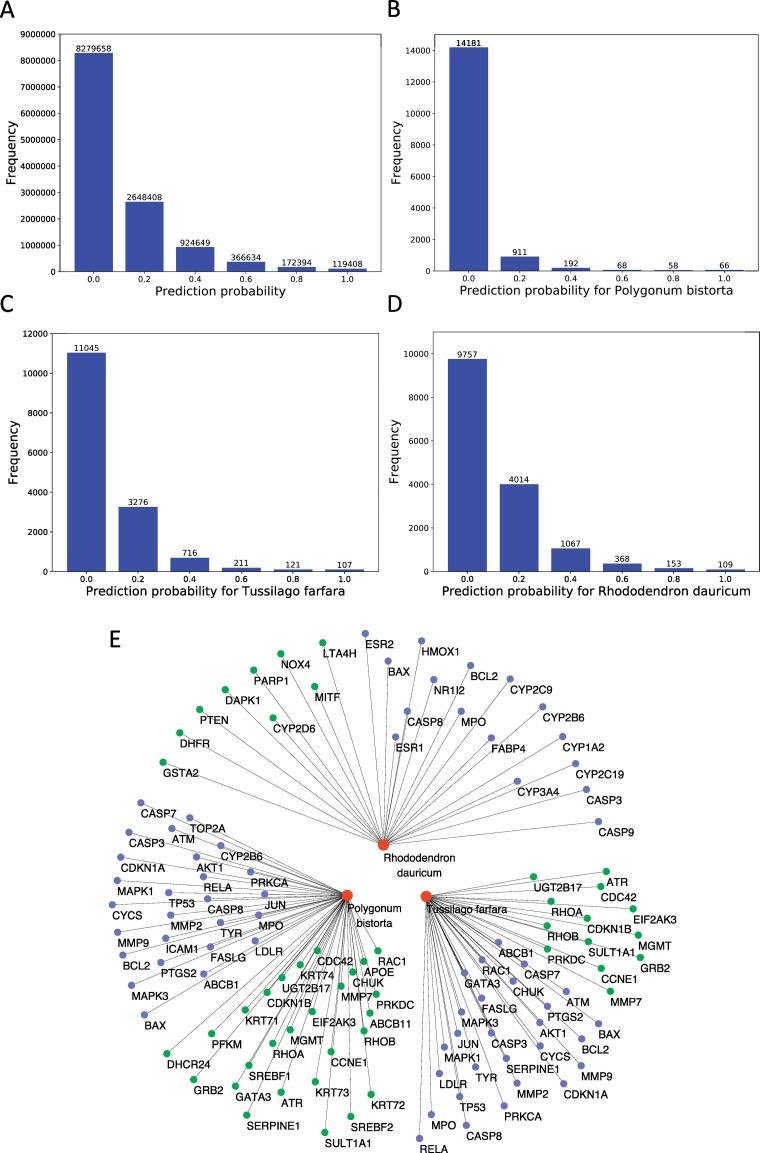
Prediction of novel herb-target interaction. The distributions of the prediction probability of all unknown herb-target pairs **(A)**, *Polygonum bistorta***(B)**, *Tussilago farfara***(C)** and *Rhododendron dauricum***(D)**. **(E)** The herb-target interaction network of *Polygonum bistorta*, *Tussilago farfara* and *Rhododendron dauricum.* Herb-target interactions were collected from TCMID or literature. The herb nodes were shown in red circles, and the validated targets were marked by blue circles, while the non-validated targets were shown by green circles. (For interpretation of the references to colour in this figure legend, the reader is referred to the web version of this article.)

After confirming the reliability of HTINet, we evaluated all the remaining 12,511,151 unknown herb-target pairs, and ranked them by the probability. According to the KNN-HTINet model, we obtained the probability of predicted herb-target pair based on the number of true sample in 5 nearest samples around it. If the number of true sample was 5, the probability was 1.0, which was calculated from 5 divided by 5. Alike, when the number was 4, 3, 2, 1 and 0, the probability was 0.8, 0.6, 0.4, 0.2, and 0.0, respectively. Therefore, each predicted herb-target pair lies in a probability of 0.0, 0.2, 0.4, 0.6, 0.8 or 1.0. As shown in Fig. 3A more than 95% herb-target pairs have prediction probabilities <0.5, indicating most pairs have no interaction. For each herb, there are averagely 814 predicted targets with probability >.5. When the threshold is set as 0.9, this number declines to 148.

Next, we randomly selected three herbs, *Polygonum bistorta*, *Tussilago farfara* and *Rhododendron dauricum*, and predicted their targets. Consistent with the overall results, the distribution of prediction probability for these herbs showed that most predicted herb-target pairs have prediction probability <.5 (98.76%, 97.16% and 95.93% for *Polygonum bistorta*, *Tussilago farfara* and *Rhododendron dauricum*, respectively, Fig. 3B, C and D). Then, the predicted results of the three herbs (Table S4) were compared with the herb-target interaction data curated in TCMID [43], which is a comprehensive TCM database containing herb-ingredient-target interaction data collected from literatures. As shown in Fig. 3E we found that 47.1% predicted targets for *Polygonum bistorta*, 67.5% for *Tussilago farfara* and 64.0% for *Rhododendron dauricum* were validated in TCMID. Furthermore, we examined the predicted targets in literatures. Our new predictions showed that *Polygonum bistorta* can also act on TOP2A (probability = .8) and CYP2B6 (probability = .6). This new prediction can be supported by a previous study that showed that aqueous extract of *Polygonum bistorta* could significantly regulate the expression levels of the two targets [44]. Our new predictions showed that *Salvia miltiorrhiza Bunge* (Danshen in Chinese), can act on CD40 (probability = 1.0), TRPC1 (probability = .6), CRP (probability = .6) and TIMP (probability = .6). These new predictions can be supported by the previous study which showed that *Salvia miltiorrhiza Bunge* could downregulate the levels of the four targets, contributing to inhibition of atherosclerosis [45]. *Panax notoginseng* (Sanqi in Chinese) was predicted to target VCAM1 (probability = 1.0) and ICAM1 (probability = 1.0). The two targets were proved to be reduced by *Panax notoginseng* treatment in a previous study [46]. *Panax ginseng* (Renshen in Chinese) has been proved to protect against amyloid β-induced neurotoxicity by acting on DSTN and TOMM40 relate to actin cytoskeleton organization [47]. Consistently, HTINet displayed the probability of *Panax ginseng* targeting the two proteins >0.5 (probability of DSTN = 0.8 and probability of TOMM40 = 0.6).

### 3.5 Case Study Using Gene Expression Omnibus (GEO) Database

Furthermore, we conducted the case study according to Gene Expression Omnibus (GEO) database [48]. The GEO is an international public repository for high-throughput microarray and next-generation sequence functional genomic data sets submitted by the research community [49].

Previous studies have reported the anti-tumor effects of Tanshinone IIA (Tan IIA), which is extracted from the root of *Salvia miltiorrhiza* (Danshen in Chinese), on various human cancer cells. Then, we want to explore which genes in cells are specifically affected. Therefore, we conducted an analysis of gene expression underlying Tan IIA's apoptotic effects on leukemia cells using a GEO data set (GSE33358). We used online analysis tool GEO2R, obtaining the top 10 differentially expressed genes (Table S5) and the prediction probability of these genes in HTINet were also shown in Table S5. We found the probability of 60.0% genes in the top 10 differentially expressed genes >0.5.

Next, we also conducted an analysis of gene expression in normal lung and diabetic lung using a GEO data set (GSE15900) to find out the impact of diabetes on the cellular and molecular processes in the lung, obtaining the top 10 differentially expressed genes (Table S6). In addition, *Panax ginseng* (Renshen in Chinese) could be used for the treatment of diabetes. Therefore, we calculated the prediction probability of these differentially expressed genes in HTINet (Table S6). The results showed that the probability of 60.0% genes in the top 10 differentially expressed genes >0.5.

Finally, we conducted an analysis of gene expression in animals with epilepsy and animals without epilepsy using a GEO data set (GSE27166) to find out the impact of epilepsy, obtaining the top 10 differentially expressed genes (Table S7). Furthermore, *Pueraria* (Gegen in Chinese) could be used for the treatment of epilepsy. Therefore, we calculated the prediction probability of these differentially expressed genes in HTINet (Table S7). The results showed that the probability of 40.0% genes in the top 10 differentially expressed genes >0.5.

## 4 Discussion

Herbal medicine differs in substance, methodology and philosophy from modern medicine. The applications of herbal medicine are mostly derived from the accumulation of empirical evidence and perception. Compared with modern medicine, the most distinguishing feature of herbal medicine is that an herbal preparation is a mixture of numerous chemical ingredients [6]. Although researches have applied a series of chemical analysis methods to disassemble the complexity of herbal medicine. The underlying mechanisms of most herbal preparations are still obscure for some reasons. First, most medicinal herbs contain many ingredients and it is difficult to differentiate between effective and non-effective. In addition, even the active compounds have been identified, their corresponding pharmacological targets also need to be discovered. All of this work is a slow and troublesome process even if the computational ligand-based prediction methodology was utilized. These concerns prompted us to consider an alternative strategy that might be capable of detecting the pharmacological targets by detouring around the complexity of herbal medicine. Previously, we proposed a transcriptome-based inference approach to identify the targets of herbal medicine [50]. This method assumes that drugs with the similar biological mechanisms will have similar gene expression profiles. Thus, if knowing the transcriptional profile of a query herbal preparation, its targets can be assessed by comparing its transcriptional profile with that of drugs with known targets [51]. However, this method needs the transcriptional profile of herbal medicine firstly being measured and also is restricted by the database size of the transcriptome data of drugs with known targets. Here, we developed a network-based embedding representation method HTINet to infer novel herb-target interactions.

Network-based methods explore the latent correlation features of different network nodes to predict their interactions, and have become a popular tool for drug discovery and repositioning [14]. A network-based approach is generally a scalable framework that can integrate heterogeneous data sources that can improve the performance of drug-target interactions from a multi-view perspective. For example, Yunan et al. integrated diverse drug-related information, including drugs, proteins, diseases and side-effects into a heterogeneous network to predict drug-target interactions, which achieves substantial performance improvement over other state-of-the-art methods [15]. In the present work, to boost the accuracy of herb-target interaction prediction, HTINet has incorporated different types of information including TCM information (e.g. herbal efficiency), TCM clinical information (e.g. herb-disease/symptom association) and genomic data (e.g. protein-protein interactions).

The main challenge in network-based methods comes from the complexity and heterogeneity of datasets. Generally, network propagation methods, such as random walk [52], information diffusion [53] and electrical resistance [54], have been used to amplify the network information. For example, we have constructed a heterogeneous herb-target network to identify candidate targets for herbs by using the random walk algorithm [21]. However, the method considered only unilateral similarity of herbs or targets likely loses substantial network information. In addition, directly using the diffusion states as the features might easily suffer from the bias induced by the noise and high-dimensionality of biological data. Here, HTINet copes with the noisy, incomplete and high-dimensional nature of large-scale biological data by combining the random walk algorithm and a shallow neural network to train and extract informative representations of nodes in the network [22]. The results showed that HTINet performs better than the diffusion algorithm PRINCE in the heterogeneous network.

In the future, our work has three further directions to improve the ability of HTINet. Firstly, we will include more related data in our model, such as gene expression, pathway and Gene Ontology (GO) information. Meanwhile, we would try the drug-drug similarity according to the OFFSIDES database [55] in our future work. In addition, as the HTINet model doesn't conflict with other target prediction approaches including ligand-based and transcriptome-based methods, we plan to integrate all these methods together to help us predict the novel herb-target interactions. Also, since the current sample size is limited, next we would expand our sample size to try other deep learning algorithms in our future works. Finally, we will choose some suitable ways to select gold positive and negative datasets in future work to improve the performance of HTINet.

## Author Contributions

XZZ, PL and JXC conceived and designed the study. NW and PL analyzed data and wrote the manuscript; XCH and KY collected related data; YHP, BYL, QZ, RSZ, ZYG and HX reviewed the methods and the results. All authors have reviewed and revised the manuscript.

## Funding

The work is supported by the National Key Research and Development Program (2017YFC1703506, 2017YFC1700106), the Fundamental Research Funds for the Central Universities (2017YJS057, 2017JBM020), the Special Programs of Traditional Chinese Medicine (201407001, JDZX2015170 and JDZX2015171), the National Natural Science Foundation of China (81703945), and the National Key Technology R&D Program (2013BAI02B01 and 2013BAI13B04).

## Conflict of Interest

The authors declare that they have no conflicts of interest.

## Appendix A Supplementary data

Supplementary material Image 1
