# Supplementary material for: Herb Target Prediction Based on Representation Learning of Symptom related Heterogeneous Network
Source: Comput Struct Biotechnol J. 2019 Feb 8;17:282–90. doi: 10.1016/j.csbj.2019.02.002 (PMC6396098; doi:10.1016/j.csbj.2019.02.002)
Supplement: Supplementary file 1 — Supplementary material [file mmc1.docx]

Supplementary Material

Herb Target Prediction Based on Representation Learning of Symptom related Heterogeneous Network

# Supplementary Tables

**Table S1. Key resources.**

| **Name** | **Composition** | **Source** |
| --- | --- | --- |
| Herb-target associations | 310 herbs, 1065 targets and 8933 herb-target links | HIT |
| Herb-efficacy associations | 829 herbs, 373 efficacies and 3830 herb-efficacy links | CHPA |
| Herb-symptom associations | 1812 herbs, 143 symptoms and 7982 herb-symptom links | CHPA |
| Herb-disease associations | 2019 herbs, 1032 diseases and 23451 herb-disease links | CHPA |
| Drug-symptom associations | 893 drugs, 1116 symptoms and 4864 drug-symptom links | SIDER |
| Drug-disease associations | 1170 drugs, 1763 diseases and 12821 drug-disease links | SIDER |
| Drug-target interactions | 1581 drugs, 1635 targets and 7077 drug-target interactions | DrugBank |
| drug ATC classification | 1456 drugs, 1997 categories and 2035 drug-category links | KEGG |
| Drug-drug associations | 1354 drugs and 4481 links | Drugs linked with similar ATC |
| Herb-herb associations | 809 herbs and 15133 links | Herbs linked with similar efficacy |
| Protein-protein interactions | 15553 proteins and 319889 interactions | String10 |
| Disease-symptom associations | 13721 diseases, 3309 symptoms and 72248 disease-symptom links | MalaCards |
| Symptom-symptom associations | 421 symptoms and 476 links | SemMedDB |
| Disease-disease associations | 7042 diseases and 18418 links | SemMedDB |

**Table S2. The performance of PRINCE and HTINet for herb-target interaction prediction.**

| **Model** | **AUROC** | **AUPR** |
| --- | --- | --- |
| PRINCE | 67% | 88% |
| DT-HTINet | 61% | 57% |
| RF-HTINet | 75% | 71% |
| LR-HTINet | 77% | 76% |
| SVM-HTINet | 80% | 79% |
| GBDT-HTINet | 83% | 83% |
| KNN-HTINet | 95% | 94% |
| Artificial Neural Network | 82% | 79% |

**Table S3. The performance of HTINet for herb-target interaction prediction whose drug-drug similarities are calculated by Jaccard.**

| **Model** | **AUROC** | **AUPR** |
| --- | --- | --- |
| DT-HTINet | 66% | 74% |
| RF-HTINet | 76% | 76% |
| LR-HTINet | 82% | 81% |
| SVM-HTINet | 81% | 80% |
| GBDT-HTINet | 85% | 85% |
| KNN-HTINet | 91% | 93% |
| Artificial Neural Network | 85% | 85% |

**Table S4. Prediction probabilities of herb-target pairs.**

| **Herb** | **Ingredient** | **Target** | **Probabilities** |
| --- | --- | --- | --- |
| Polygonum bistorta | gallicacid | CDKN1A | 1.0 |
| Polygonum bistorta | gallicacid | BAX | 1.0 |
| Polygonum bistorta | gallicacid | PTGS2 | 1.0 |
| Polygonum bistorta | gallicacid | TYR | 1.0 |
| Polygonum bistorta | gallicacid,  β-sitosterol | CASP3 | 1.0 |
| Polygonum bistorta | gallicacid | AKT1 | 1.0 |
| Polygonum bistorta | gallicacid | RELA | 1.0 |
| Polygonum bistorta | gallicacid | FASLG | 1.0 |
| Polygonum bistorta | gallicacid | BCL2 | 1.0 |
| Polygonum bistorta | gallicacid | LDLR | 1.0 |
| Polygonum bistorta | gallicacid | CASP8 | 1.0 |
| Polygonum bistorta | gallicacid | MMP2 | 1.0 |
| Polygonum bistorta | gallicacid | MMP9 | 1.0 |
| Polygonum bistorta | gallicacid | TP53 | 1.0 |
| Polygonum bistorta | gallicacid | MAPK1 | 1.0 |
| Polygonum bistorta | β-sitosterol | ICAM1 | 1.0 |
| Polygonum bistorta | gallicacid | MAPK3 | 0.8 |
| Polygonum bistorta | gallicacid | JUN | 0.8 |
| Polygonum bistorta | gallicacid | MPO | 0.8 |
| Polygonum bistorta | gallicacid | ATM | 0.8 |
| Polygonum bistorta | gallicacid | CYCS | 0.8 |
| Polygonum bistorta | gallicacid | CASP7 | 0.8 |
| Polygonum bistorta | gallicacid | ABCB1 | 0.6 |
| Polygonum bistorta | gallicacid | PRKCA | 0.6 |
| Polygonum bistorta | gallicacid | SERPINE1 | 0.4 |
| Polygonum bistorta | gallicacid | GATA3 | 0.4 |
| Polygonum bistorta | β-sitosterol | SREBF2 | 0.4 |
| Polygonum bistorta | β-sitosterol | SREBF1 | 0.4 |
| Polygonum bistorta | amylum | PFKM | 0.2 |
| Polygonum bistorta | gallicacid | SULT1A1 | 0.2 |
| Polygonum bistorta | gallicacid | RHOA | 0.2 |
| Polygonum bistorta | gallicacid | CDC42 | 0.2 |
| Polygonum bistorta | gallicacid | RAC1 | 0.2 |
| Polygonum bistorta | amylum | KRT74 | 0.0 |
| Polygonum bistorta | amylum | KRT71 | 0.0 |
| Polygonum bistorta | amylum | KRT72 | 0.0 |
| Polygonum bistorta | amylum | KRT73 | 0.0 |
| Polygonum bistorta | gallicacid | PRKDC | 0.0 |
| Polygonum bistorta | gallicacid | CDKN1B | 0.0 |
| Polygonum bistorta | gallicacid | UGT2B17 | 0.0 |
| Polygonum bistorta | gallicacid | RHOB | 0.0 |
| Polygonum bistorta | gallicacid | CCNE1 | 0.0 |
| Polygonum bistorta | gallicacid | ATR | 0.0 |
| Polygonum bistorta | gallicacid | MGMT | 0.0 |
| Polygonum bistorta | gallicacid | CHUK | 0.0 |
| Polygonum bistorta | gallicacid | GRB2 | 0.0 |
| Polygonum bistorta | gallicacid | MMP7 | 0.0 |
| Polygonum bistorta | gallicacid | EIF2AK3 | 0.0 |
| Polygonum bistorta | β-sitosterol | DHCR24 | 0.0 |
| Polygonum bistorta | β-sitosterol | APOE | 0.0 |
| Polygonum bistorta | β-sitosterol | ABCB11 | 0.0 |
| Tussilago farfara | gallicacid | PRKCA | 1.0 |
| Tussilago farfara | gallicacid | CDKN1A | 1.0 |
| Tussilago farfara | gallicacid | BAX | 1.0 |
| Tussilago farfara | gallicacid | PTGS2 | 1.0 |
| Tussilago farfara | gallicacid | TYR | 1.0 |
| Tussilago farfara | gallicacid | CASP3 | 1.0 |
| Tussilago farfara | gallicacid | JUN | 1.0 |
| Tussilago farfara | gallicacid | AKT1 | 1.0 |
| Tussilago farfara | gallicacid | RELA | 1.0 |
| Tussilago farfara | gallicacid | FASLG | 1.0 |
| Tussilago farfara | gallicacid | BCL2 | 1.0 |
| Tussilago farfara | gallicacid | MPO | 1.0 |
| Tussilago farfara | gallicacid | LDLR | 1.0 |
| Tussilago farfara | gallicacid | CASP8 | 1.0 |
| Tussilago farfara | gallicacid | MMP2 | 1.0 |
| Tussilago farfara | gallicacid | CYCS | 1.0 |
| Tussilago farfara | gallicacid | MMP9 | 1.0 |
| Tussilago farfara | gallicacid | TP53 | 1.0 |
| Tussilago farfara | gallicacid | MAPK1 | 1.0 |
| Tussilago farfara | gallicacid | CASP7 | 1.0 |
| Tussilago farfara | gallicacid | MAPK3 | 0.8 |
| Tussilago farfara | gallicacid | RAC1 | 0.8 |
| Tussilago farfara | gallicacid | ATM | 0.8 |
| Tussilago farfara | gallicacid | ABCB1 | 0.6 |
| Tussilago farfara | gallicacid | SERPINE1 | 0.6 |
| Tussilago farfara | gallicacid | GATA3 | 0.6 |
| Tussilago farfara | gallicacid | CHUK | 0.6 |
| Tussilago farfara | gallicacid | PRKDC | 0.2 |
| Tussilago farfara | gallicacid | SULT1A1 | 0.2 |
| Tussilago farfara | gallicacid | RHOA | 0.2 |
| Tussilago farfara | gallicacid | CDC42 | 0.2 |
| Tussilago farfara | gallicacid | UGT2B17 | 0.2 |
| Tussilago farfara | gallicacid | ATR | 0.2 |
| Tussilago farfara | gallicacid | MGMT | 0.2 |
| Tussilago farfara | gallicacid | CDKN1B | 0.0 |
| Tussilago farfara | gallicacid | RHOB | 0.0 |
| Tussilago farfara | gallicacid | CCNE1 | 0.0 |
| Tussilago farfara | gallicacid | GRB2 | 0.0 |
| Tussilago farfara | gallicacid | MMP7 | 0.0 |
| Tussilago farfara | gallicacid | EIF2AK3 | 0.0 |
| Rhododendron dauricum | syringicacid | MPO | 1.0 |
| Rhododendron dauricum | scopoletin,  grifolin | CASP3 | 1.0 |
| Rhododendron dauricum | isoimperatorin | HMOX1 | 1.0 |
| Rhododendron dauricum | grifolin | BAX | 1.0 |
| Rhododendron dauricum | grifolin | CASP9 | 1.0 |
| Rhododendron dauricum | grifolin | BCL2 | 1.0 |
| Rhododendron dauricum | grifolin | CASP8 | 1.0 |
| Rhododendron dauricum | germacrone | CYP1A2 | 1.0 |
| Rhododendron dauricum | germacrone | CYP3A4 | 1.0 |
| Rhododendron dauricum | germacrone | NR1I2 | 1.0 |
| Rhododendron dauricum | dihydroresveratrol | ESR2 | 1.0 |
| Rhododendron dauricum | dihydroresveratrol | ESR1 | 1.0 |
| Rhododendron dauricum | germacrone | CYP2C9 | 0.8 |
| Rhododendron dauricum | germacrone | CYP2B6 | 0.8 |
| Rhododendron dauricum | germacrone | CYP2C19 | 0.6 |
| Rhododendron dauricum | dihydroresveratrol | FABP4 | 0.6 |
| Rhododendron dauricum | isoimperatorin | GSTA2 | 0.4 |
| Rhododendron dauricum | gossypetin | PTEN | 0.4 |
| Rhododendron dauricum | germacrone | CYP2D6 | 0.4 |
| Rhododendron dauricum | grifolin | DAPK1 | 0.2 |
| Rhododendron dauricum | gossypetin | PARP1 | 0.2 |
| Rhododendron dauricum | dihydroresveratrol | LTA4H | 0.2 |
| Rhododendron dauricum | syringicacid | DHFR | 0.0 |
| Rhododendron dauricum | scopoletin | MITF | 0.0 |
| Rhododendron dauricum | azaleatin | NOX4 | 0.0 |

**Table S5. Top 10 differentially expressed genes in GSE33358.**

| **Gene** | **Rank** | **Probability in HTINet** | **Rank in HTINet** |
| --- | --- | --- | --- |
| CRTAM | 1 | 0.6 | 1920/15428 |
| NEAT1 | 2 | -- | -- |
| LINC00622 | 3 | -- | -- |
| PPP2R2B | 4 | 0.6 | 1920/15428 |
| CATSPER3 | 5 | 0.4 | 4055/15428 |
| FIGF | 6 | 1.0 | 1/15428 |
| BUB1 | 7 | 0.6 | 1920/15428 |
| NMRAL1P1 | 8 | -- | -- |
| RAB11FIP4 | 9 | 0.6 | 1920/15428 |
| HTR2B | 10 | 0.8 | 600/15428 |

**The symbol “--” represents the gene is not included in our datasets**

**Table S6. Top 10 differentially expressed genes in GSE15900.**

| **Gene** | **Rank** | **Probability in HTINet** | **Rank in HTINet** |
| --- | --- | --- | --- |
| ARRDC4 | 1 | 0.6 | 4783/15428 |
| CTGF | 2 | 1.0 | 1/15428 |
| RARRES2 | 3 | 0.4 | 8927/15428 |
| NR1D1 | 4 | 0.6 | 4783/15428 |
| NR1D2 | 5 | 0.6 | 4783/15428 |
| ENPEP | 6 | 0.8 | 1741/15428 |
| P2RX1 | 7 | 0.2 | 12739/15428 |
| TP53INP2 | 8 | 1.0 | 1/15428 |
| BSG | 9 | 0.4 | 8927/15428 |
| ETV3 | 10 | 0.2 | 12739/15428 |

**The symbol “--” represents the gene is not included in our datasets**

**Table S7. Top 10 differentially expressed genes in GSE27166.**

| **Gene** | **Rank** | **Probability in HTINet** | **Rank in HTINet** |
| --- | --- | --- | --- |
| KIF15 | 1 | 0.2 | 11596/15428 |
| IGFBPL1 | 2 | -- | -- |
| TSPAN8 | 3 | 0.8 | 1816/15428 |
| IGF2R | 4 | 0.8 | 1816/15428 |
| HTRA1 | 5 | 1.0 | 1/15428 |
| RHD | 6 | 0.2 | 11596/15428 |
| RAB23 | 7 | 0.6 | 4669/15428 |
| KIF23 | 8 | 0.2 | 11596/15428 |
| PSMA1 | 9 | 0.2 | 11596/15428 |
| ARMT1 | 10 | -- | -- |

**The symbol “--” represents the gene is not included in our datasets**
